# Healthcare Professionals’ Perspectives on Improving Family-Centred Pain Care in a Tertiary Pediatric Centre

**DOI:** 10.1177/08445621241228063

**Published:** 2024-01-22

**Authors:** Elise Kammerer, Joelle Fawcett-Arsenault, Lexyn Iliscupidez, Samina Ali

**Affiliations:** 1 Department of Pediatrics, Faculty of Medicine & Dentistry, Women and Children's Health Research Institute, Faculty of Medicine & Dentistry, 3158 University of Alberta, Edmonton, Alberta, Canada; 2 Patient and Family Centred Care, Stollery Children's Hospital, Alberta Health Services, Edmonton, Alberta, Canada; 3 Department of Biological Sciences, University of Alberta, Edmonton, Alberta, Canada; 4 Department of Pediatrics, Faculty of Medicine & Dentistry, Women and Children's Health Research Institute, Faculty of Medicine & Dentistry, 3158 University of Alberta, Edmonton, Alberta, Canada

**Keywords:** Children, patient- and family-centered care, pain management, nursing, qualitative research

## Abstract

**Background:**

Despite being a core component of family-centered and compassionate care, children's pain is often undertreated in Canadian hospitals. Nurses’ and other healthcare professionals’ (HCPs) ability to understand and respond to a child and their family's pain care needs is integral to improving this care in a family-centered manner.

**Purpose:**

To understand nurses’ and other HCPs’ perceptions of child and family needs to make care more collaborative and patient- and family-centered.

**Methods:**

Eighteen participants were recruited and represented the specialties of nursing (*n* = 8), psychology (*n* = 1), child life services (*n* = 2), medicine/surgery (*n* = 3), and administration/leadership (*n* = 4); 3 of the administrators had a nursing background. Transcripts were analysed using a semantic, inductive approach with two coders using a codebook to ensure reliability.

**Results:**

Participants felt that pain care was important, but that it needs to take greater priority in the hospital. In our analysis, we identified four core needs that nurses and other HCPs have to provide better pain care: 1. Better acknowledgement of child and family experiences; 2. Better visual and written knowledge translation tools for patients and families; 3. Better provision of verbal pain education to children and families by nurses and other HCPs; and 4. Help for patients and families to advocate for better pain care when they feel their needs are not being met.

**Conclusions:**

Nurses and other HCPs value patient- and family-centered pain care, and wish to empower families to advocate for it when it is sub-optimal.

## Introduction

“Family-centred children's pain care should revolve around finding out what families need and what they what they want at every level… medically, physically, socially, psychologically… and working with families where they are, given their understanding of pain, their understanding of the health care system. Sometimes, actually in very early stages, it's about helping them understand how the healthcare system works in our institution and in the area” (Participant 14).

All children will experience painful procedures in their childhood, whether as part of newborn metabolic screening, routine immunizations, or requiring a skin-breaking procedure for health care investigations. Additionally, between 11 and 38 per cent of children and youth will experience chronic pain (Neville et al., 2020). Despite this, children's pain management in Canadian hospitals remains suboptimal (Ali et al., 2016; Gates et al., 2018; Shave et al., 2018; Stevens et al., 2011). Although there is an ever-growing body of literature on how to best manage children's pain, this knowledge is often slow to be put into practice (Chambers, 2018).

When nurses and other healthcare professionals (HCPs) effectively integrate caregivers and children as part of their care team, they are able to provide higher-quality and more timely care (Matziou et al., 2018). We now know that family-centered pain care must be a priority when providing care for children (Byczkowski et al., 2016), as inadequate pain management can result in poorer outcomes and extended hospital stays (von Baeyer et al., 2004; Young, 2005). Seeing their child experience preventable pain can also be significantly distressing for caregivers (Smith et al., 2007); further if caregivers are unsure how to best to support their child during these stressful times, their child's distress during current and future procedures can increase (Moline et al., 2021). When care is family-centered, children's, caregivers’, and nurses’ and other HCPs’ overall satisfaction with their care experience improves (Byczkowski et al., 2016).

Nurses and other HCPs play an important role in family-centered pain care, by either facilitating or restricting a family's involvement in their child's pain management (Govindaswamy et al., 2022). For example, nurses can educate children and their caregivers about pain management strategies and encourage both child empowerment and direct caregiver involvement in pain care. Alternatively, if nurses and other HCPs do not accurately assess families’ needs, they may stand in the way of caregivers who want to help manage their child's pain (Govindaswamy et al., 2022). This study sought to understand nurses’ and other HCPs’ needs to promote patient- and family-centered pain care needs at a Canadian tertiary pediatric center. An understanding of nurse and HCP needs in this area can facilitate the broad promotion of patient- and family-centered care and improved integration of patients and families into the care team.

## Methods

### Setting

This sub-study of an existing qualitative data set on HCPs’ perspectives on pain management was conducted at the Stollery Children's Hospital, a tertiary pediatric center located in Edmonton, Alberta, Canada. The facility has 218 beds and is a national leader in pediatric cardiac surgery and organ transplantation (Alberta Health Services, n.d.). The hospital has a facility-wide pain management committee, an acute pain service that works to treat complex pain for inpatients, and an outpatient chronic pain clinic. The interviews for this study were conducted as part of a local quality improvement initiative, and these data therefore received formal exemption from ethics approval from the University of Alberta's Research Ethics Board.

### Interview guide development and participant recruitment

A guide for semi-structured interviews was co-developed by members of the study team (EK, SA), clinical staff, and a person with lived experience of pain. Interviews explored themes of pain management at the hospital and how patients and families can be better centered in children's pain care. Participants were recruited through snowball sampling (Green & Thorogood, 2018; Merriam & Tisdell, 2016) by first recruiting interested members of the hospital's pain management committee. Interviews were conducted via online and in-person interviews during the Fall of 2020 by a study team member (EK), who is a Caucasian female knowledge mobilization specialist. Interviews lasted between 25 to 75 min. Verbatim transcripts were produced, anonymized, and analyzed using MAXQDA2020. Participants were given a $25 electronic gift card as a token of appreciation.

### Analyses

The data were coded using an inductive, semantic approach in order to identify salient themes identified by participants in their interviews (Braun & Clarke, 2006). This coding used a codebook, whereby themes were selected as summaries for different topics, with inclusion and exclusion criteria being noted in the codebook (Braun & Clarke, 2021; Guest et al., 2012). Primary coding was completed by one team member (EK), with a second team member (LI) coding a portion of the dataset to ensure reliability. This approach to coding identified the various areas of pain management practice discussed by participants including pain management strategies, pain assessment, family-centered care, advocacy, resources for clinicians and families, and evidence-based practice.

## Results

Eighteen participants were recruited and represented the specialties of nursing (*n* = 8), psychology (*n* = 1), child life services (*n* = 2), medicine/surgery (*n* = 3), and administration/leadership (*n* = 4); 3 of the administrators had a nursing background. 94% (*n* = 17) of the participants were female. Hospital services represented by the participants included the emergency department, acute and chronic pain services, oncology, hematology, palliative care, and peri-operative units.

We identified four main themes that define nurse and HCP needs in order to better support family-centered pediatric pain care: 1. Better acknowledgement of child and family experiences; 2. Better visual and written knowledge translation tools for patients and families; 3. Better provision of verbal pain education to children and families by nurses and other HCPs; and 4. Help for patients and families to advocate for better pain care when they feel their needs are not being met.

### 1. Acknowledging patient and family's pain experiences

*“I think just asking open-ended questions… ‘What is your experience? How do you feel pain control has been for the child?’ Of course, depending on the child's age, you might be involving them, too in the discussions… ‘Is your pain well controlled? Could it be better controlled? What could we do?*’” (Participant 13).

Ideally, children's experience of pain and their family's reaction to those experiences should be accepted and validated by all nurses and other HCPs. Unfortunately, participants noted that patients’ and families’ experiences of pain are often minimized or misunderstood by nurses and other HCPs from many disciplines at the hospital. One participant noted that, when a child's pain experience is poorly understood, this not only negatively impacts patient- and family-centered pain care, but the child's healing, as well:

“*I think the barriers that are faced are day-to-day … the under-recognition or appreciation of pain in children. Just either by nursing staff, physicians… a variety of professionals… and our desire always to treat with the minimal required medication sometimes leads to the under-treatment of pain, which can inhibit recovery”* (Participant 13).

Other participants remarked that they frequently observe children's and families’ pain experience being minimized or ignored by nurses and other HCPs, resulting in potentially traumatic situations for the child. For example, even though the hospital has several areas which do not support the use of physical restraint, it is still occasionally used for children who resist medical procedures:

“*I hear from the staff that they were just pinning her down and she was just screaming her head off… Or they poked the kid and didn't have time for Maxilene … I don't get it, because in the end you got your job done, but the repercussions of what happens later… the anger, the lack of trust, the sadness, and just the idea that you could have done something better for a child and you didn't take the time”* (Participant 15).

Participants noted the necessity of listening to children and families about their pain experiences. Acknowledging these experiences is integral to providing patient- and family-centered pain care.

### 2. Written knowledge mobilization tools

*“When we look at family-centred care, children live with families who don’t live in hospitals… hopefully we are episodic in their journey… [we need to] bring them the evidence.”* (Participant 1)

When presenting to a hospital for care, whether in an emergency or for a planned surgery/visit, participants highlighted that most families do not know what pain management strategies are available to them. Participants noted that sharing knowledge mobilization (KM) tools such as posters, pamphlets, or visual reminders with families is one method to achieve this. While KM efforts are often focused on clinicians, patients and families can benefit from such efforts, as well. For example, some participants mentioned the utility of posters at the bedside that inform patients and families that they may ask for topical anesthetic before a skin-breaking procedure: “*Putting up those posters asking and reminding parents that they could ask for the numbing cream*” (Participant 1). These posters and other KM tools can be simple, such as “reminders [that] ‘*If you have pain, tell your nurse*,’ (Participant 11) and can help empower caregivers to advocate for better pain care for their child, and support nurses and other HCPs in delivering better care.

Some participants mentioned that they sometimes have difficulty practicing in an evidence-informed way when families have sought resources on their own outside of the hospital, which may make recommendations that are not evidence-informed. When discussing online networks for children and youth with chronic pain, one participant mentioned that difficulties can arise:

“*We have to be careful with some of the networks because there isn't a lot of oversight… I think in many cases, those networks are organized and driven by really excellent parents and caring people who sometimes are well-intentioned but are accessing information sources that come from places that maybe aren't necessarily best practice*” (Participant 14).

An important issue regarding KM and knowledge about evidence-informed pain management was highlighted in our interviews: children and families actively seek this information from other sources, as they often do not receive sufficient information in their healthcare setting. Yet nurses and other HCPs do not always find that the information that children and families present to reflect current best evidence. Participants shared that this potential conflict of information suggests that there is a need for making better evidence-based KM tools easily accessible for patients and families.

### 3. Verbal pain education for patients and families

*“I think the frequent flyers know better. They come back and they go, ‘I need Maxilene.’ But if someone comes in for the first time, they don't know what to ask for… they don't know and they need to know”* (Participant 15).

In their interviews, participants spoke about the need for nurses and other HCPs to educate families and children if the child was old enough and cognitively able to understand shared information. As mentioned above, families who often receive care at the hospital are usually aware of the different pain management strategies available for their child. Families less experienced with receiving care in the hospital may be unaware of different strategies, such as the availability of topical anesthetic or that nurses can instruct caregivers on how to perform comfort positioning with their child. As Participant 4 mentioned, “*If you never know it's an option, you're not going to be able to ask for it*.”

When patients and families do receive education, this education is usually provided verbally and just prior to or during an admission or procedure. For example, some children visit the hospital preoperatively before major surgeries to speak with the anesthesiologist, and the team will “*integrate some pain management teaching as part of the appointment*” (Participant 12). Nurses and other HCPs reported doing their best to make sure education was multimodal and reflected the “3Ps” of pain management (physical, psychological, and pharmacologic strategies):

“*I go over the medications and how they're being used together, for what purpose, and what the anticipated timeline is. Let's say it's an orthopedic patient… we review elevating the limb, repositioning… then we go over… What do you usually like to do? Do they have music on their phone? Do they like watching things?”* (Participant 11).

While participants recognized that multimodal pain care was important, nurses and other HCPs highlighted that there tends to be a stress on only pharmacologic pain management when verbally educating families:

“*A big part of my teaching is telling them… when it's starting to hurt, take something. Don't wait until that pain is stopping you from getting up getting moving, doing all the post-op things we need you to do… take something before that”* (Participant 12).

Some participants mentioned that there needs to be continued improvement at the hospital to make children's pain experiences truly patient- and family-centered with a focus on respect and dignity, information sharing, collaboration, and joint decision-making. To aid in retention, nurses and other HCPs should consider sharing information in a variety of formats: “*[Education] really involves having parents tell us how they best would like to receive that information”* (Participant 1). Relying primarily on verbal pain education at the hospital may mean that patients and families are not receiving their education in a multimodal way which may best support their learning, affect their ability to retain or recall the information, and therefore their confidence to advocate for it when it is acutely needed.

### 4. Enabling patient and family advocacy through pain education

*“We saw a mom and an infant in follow-up recently… and the baby had EMLA on, and the mom was asking about that… and it was this light-bulb moment for her, and she said, ‘Well, how can it be that he's never had this before? If I knew this existed, I would have asked for it… think of all the painful pokes he's had where he didn't have this.’ I know she'll ask for it, going forward”* (Participant 4).

Participants felt that families can and should take an active role in their child's pain management experience, although families’ role can vary based on their knowledge and comfort in advocating for pain care for their child. Participants shared that KM tools and point-of-care education are important components that can help families and patients feel more comfortable to do so:

“*I think it depends on the child and where they're at, and also the parent. I think parents can become quite involved in developing and implementing pain management strategies, so we get the non-pharmacologic stuff, the distraction… but I think they have to be taught those skills”* (Participant 4).

While Participant 4 highlighted that patients and families need to be taught pain management strategies to be able to advocate, they stated that this may be difficult if the only education received, if any at all, is verbal and brief.

Participants acknowledged that coming to the hospital can be “*intimidating”* (Participant 11) to families, as families “*want to help [their child, but] sometimes they’re not sure [how]”* (Participant 11). When nurses and other HCPs help educate families and ensure they have access to evidence-based resources, it can improve families’ pain experience both in the moment and into the future:

“*It can equip children and families to make requests and be a part of the care team. We need to be providing those things whether or not a family asks… I think having positive experiences also allows them to replicate them over time, whereas if it's negative, they don't necessarily know how to make it better”* (Participant 4).

When a family receives adequate pain education and has a positive experience in applying that education through advocacy, they are more likely to feel comfortable advocating for adequate pain management for their child in the future. Participants also noted that, when the child is involved in their pain management, they will also be more likely to advocate for better care.

## Discussion

When children's pain is poorly managed, they may experience a number of adverse effects, such as longer hospital stays, slower healing time, future healthcare avoidance or overuse, increased likelihood of developing chronic pain, and increased anxiety regarding painful procedures in the future (Buskila et al., 2003; Kennedy et al., 2008; von Baeyer et al., 2004; Young, 2005). As such, it is critical to identify where nurses and other HCPs need more support to enable the best possible patient- and family-centered pain care. Participants in this study acknowledged that pain management is a patient and family-centered priority for nurses and other HCPs, along with the children and families, themselves.

While patient- and family-centered pain care continues to gain acceptance among nurses and other HCPs, there is currently little research on their needs to provide patient- and family-focused pediatric pain care. In a study considering trainee physicians’ perspectives of caregivers’ needs, trainees believed children needed better pain management and caregivers desired better communication and more empathy (Govindaswamy et al., 2022). Caregivers place high importance on their child's appropriate and timely pain management, yet nurses and other HCPs still underestimate the stress that a child's pain may cause the caregiver (Govindaswamy et al., 2022). While participants in our study also acknowledged that children's pain is not always optimally managed at the hospital, more decisive was their emphasis on different forms of communication for caregivers and children: receiving knowledge translation tools and education, having their experiences being listened to and acknowledged, and being provided with opportunities to learn how to advocate for better pain care.

While families do actively seek out information on how to improve their children's pain experience, up to 75 per cent of patients report not having the information they need to take an active role in their own care (Chambers, 2018). KM is an important consideration in patient and family-centered pain care, as it serves to reduce the knowledge-to-practice gap (Barwick, 2016) through the dissemination of resources or tools. Nurses and other HCPs should play an even greater and direct role in being disseminators of this knowledge by ensuring evidence-informed KT tools, ideally co-created with patient and family partners, are available for families to aid the retention of pain management knowledge (Watson & McKinstry, 2009). The combination of nurses’ and other HCPs’ commitment to patient- and family-centered pain care, to working with patients and families as partners in care and the provision and discussion of evidence-informed pain-management tools in multiple, accessible formats (e.g., posters, text messages, email, social media) will result in pain care becoming more patient- and family-centered. Patients and families, armed with information and increased confidence, can then act as essential members of the health care team, working towards the shared goals of comfort and pain reduction.

Participants’ views in this study echoed other literature that suggests nurses and other HCPs often rely on children and families to be proactive to advocate for better pain management rather than offering a multimodal pain management approach themselves (Twycross & Collins, 2013). The presence of a caregiver can indeed improve a child's pain experience as the caregiver assesses their child's pain and helps empower their child to play an active role in their own care. Nurses and other HCPs often insist that caregivers provide important insights on their child's pain experiences and therefore should advocate (Simons et al., 2020; Vasey et al., 2019). However, a lack of role clarity and misunderstanding of child and family advocacy often means nurses and other HCPs will not elicit pain reports or believe child and family reports of pain, leading to the poor management of children's pain (Simons et al., 2020; Twycross & Collins, 2013; Vasey et al., 2019). Nurses and other HCPs may support patient- and family-centered pain care and family advocacy in theory, but often do not either fully understand or ascribe to the principles of patient- and family-centered care including empowerment, engagement, and true partnership (Vasey et al., 2019). Additionally, there is often little opportunity to practice and receive feedback in PFCC-supportive behaviors. Nurses and other HCPs may have difficulty accepting children's and families’ assessment of pain and suggestions to improve pain management. Children and families must be seen as equal members of the care team, which includes being respected, encouraged, and addressed. By first acknowledging the patients’ and families’ expertise in their own child, their previous pain experiences, and knowledge, nurses and other HCPs can best plan, then take an active role in teaching patients and families how to advocate for better care during their current or future stays.

## Limitations

Participants were primarily from the nursing and child life professions. While nurses and child life specialists are essential to children's pain management, family support from physicians and other practitioners from different specialties is also required to optimally manage children's pain. Additionally, 17 out of 18 participants were female, and may not accurately represent the views of non-female participants. Further, the experiences and viewpoints of individuals who were less in favor of bettering children's pain management were not reflected in this study as all participants voiced supporting its improvement. Finally, generalizability to other centers may be limited as this was a single-center study.

## Conclusions

Nurses and other HCPs in our study acknowledged the importance of patient- and family-centredness to improving children's pain care but emphasized that they want patients and families to take an active role in advocacy to help them provide this care. Nurses and other HCPs value patient- and family-centered pain care but need the following to provide adequate pain care: 1. Better acknowledgement of child and family experiences; 2. Better visual and written knowledge translation tools for patients and families; 3. Better provision of verbal pain education to children and families by nurses and other HCPs; and 4. Help for patients and families to advocate for better pain care when they feel their needs are not being met.
